# Ginseng: potential for the antileishmanial arsenal?

**DOI:** 10.1590/1516-3180.2013.1314517

**Published:** 2013-08-01

**Authors:** Nader Pazyar, Reza Yaghoobi

**Affiliations:** I MD. Assistant Professor, Department of Dermatology, Ahvaz Jundishapur University of Medical Sciences, Ahvaz, Iran.; II MD. Professor, Department of Dermatology, Ahvaz Jundishapur University of Medical Sciences, Ahvaz, Iran.

Cutaneous leishmaniasis (CL) develops after inoculation of the skin with parasites of the genus *Leishmania*, transmitted by phlebotomines (sandflies). Every year, CL affects approximately 1-1.5 million people worldwide, with over 90% of the cases occurring in the Middle East and South America.^1^

CL is regarded as a parasitic disease caused by *Leishmania* and no optimal medication protocol is available for this skin infection. Beneficial treatment regimens for CL should be based on experience, efficiency and the toxicity of the drugs in each region.^2^

Importantly, it has been clarified that protective immunity against CL is related to induction of Th1/Tc1 immune response, which leads to efficient parasite elimination.^3^ Interleukin (IL)-12 is unanimously believed to be a pivotal cytokine for induction of Th1/Tc1-dependent protection against *Leishmania*.^4^ Moreover, activated macrophages are the major source of IL-12, which stimulates autocrine macrophage activation.^5^

One important point needing attention is that the polyamine biosynthetic pathway is fundamental for the growth and survival of *Leishmania*. Ornithine decarboxylase (ODC) is the first enzyme in the polyamine biosynthetic pathway.^6^ Additionally, it has been recognized that ODC catalyzes the conversion of ornithine to putrescine^7^ and that the levels of putrescine are elevated in cases of resistant leishmaniasis.^8^

It has been revealed that P-glycoproteins mediate drug resistance to *Leishmania* and other protozoa and that this is followed by development of cross-resistance to numerous structurally and functionally unrelated drugs.^9^

*Leishmania* parasites are obligate intracellular organisms in mammals, and they invade macrophages and dendritic cells. Notably, it has been demonstrated that nitric oxides produced in macrophages possess a crucial role as leishmanicidal agents.^10^

Ginseng (*Panax ginseng*) has been used for thousands of years in phytomedicine and it has captured a specific position on the list of the best-selling herbal agents in the world.^11^ Ginseng modulates blood pressure, metabolism and immune functions.^12^

Interestingly, ginsenosides, which are the major active component of ginseng, have a range of biomedical effects.^13^ They are triterpene saponins, and most ginsenosides are composed of a dammarane skeleton (17 carbons in a four-ring structure) with various sugar moieties.^12^

It is noteworthy that ginseng therapy has been shown to stimulate a Th1-like immune response, which improves the course of diseases in animal models.^14^ Importantly, ginseng modulates the peripheral blood mononuclear cells and leads to higher IL-12 production. Additionally, elevated IL-12 levels can induce a more potent Th1 immune response.^15^

IH-901, a new intestinal bacterial metabolite extracted from protopanaxadiol-type ginsenosides, has been observed to suppress ornithine decarboxylase activity dose-dependently in animal skin.^16^

Protein-arginine N-methyltransferase (protein methylase I) catalyzes methylation of arginyl residues on substrate protein post-translationally. It has been found that Korean red ginseng is able to inhibit protein methylase I activity and, subsequently, polyamines *in vitro*.^17^

Lee et al. demonstrated that total saponin in ginseng and ginsenosides was capable of decreasing the putrescine levels in immobilization-stressed gerbil mice.^18^

Notably, an *in vitro* study has shown that purified Rg1 ginsenosides increase the production of nitric oxide from IFN-gamma activated macrophages.^19^

Ginsenosides have been reported to be inhibitors of P-glycoprotein (Pgp).^20^ Choi et al. explained that protopanaxatriol ginsenosides exert a chemosensitizing effect on Pgp-mediated multidrug resistance cells. Correspondingly, this component increases the intracellular accumulation of drugs through direct interaction with Pgp at the azidopine site.^21^

In summary, putting the above facts together, ginseng and ginsenosides may open up a novel therapeutic opportunity for treating cutaneous leishmaniasis. Combination of topical ginseng or ginsenosides with meglumine antimoniate might boost the therapeutic effects of this drug, increase its intracellular accumulation and, subsequently, help to reduce the resistance of parasites against it.
